# Supplementary material for: Kaizen: Decomposing cellular images with VQ-VAE
Source: PLoS One. 2025 May 30;20(5):e0313549. doi: 10.1371/journal.pone.0313549 (PMC12124493; doi:10.1371/journal.pone.0313549)
Supplement: Attachment — Submitted filename: Response to Reviewers.pdf [file pone.0313549.s001.pdf]

Response reviewer 1

We thank the reviewer for the time and effort in giving us constructive feedback. In the following we address each of the reviewer's questions:

1. We compare Kaizen to two other methods, like in the literature, and we choose Stardist and Cellpose due to wide employment by researchers and code availability. Rather than reach SOTA performance we were interested in applying generative models to segmentation. However, we will make the code available to facilitate comparison with other methodologies.

2. We apply the methodology of the same model with token parameter changes to two different datasets, which shows some generalization capability. Rather than develop a SOTA method for a large amount of data we were more interested in exploring methods to apply generative models to segmentation. However, we agree with the reviewer and future work is needed with a more powerful generative model that can benefit greatly from larger data from different datasets.

3. We have added more detail regarding error types for the U20S dataset in the results section (line 185). For the neuroblastoma dataset, the results are more balanced with 41 false positives and 44 false negatives, and might not interest the reader.

4. Our background is mainly in computer science. We are hesitant to discuss clinical applications or benefits for cell and tissue biology, given our lack of experience and shallow knowledge on the topic.

5. The other reviewer also raised this issue about sharing the code and reproducibility by other researchers. We have decided to make the code available to facilitate reproducibility by other researchers. We are still working on refactoring the code since it was very messy. We will make everything available in github (https://github.com/Danielmaj/Kaizen) and in one of the repositories recommended by PLOS ONE.

Response reviewer 2

We sincerely thank the reviewer for raising critical questions and for providing comments to improve the manuscript.

1.1 We have added in section 2.4 a more detailed description of the dataset splits. No cross validation was performed in the dataset. Through experimentation we have found that when the vq-vae model overfits the dataset the final performance of the method is severely impaired. Thus the vq-vae is trained a small amount of epochs to avoid overfitting.

1.2 The patch size was chosen such that it covers the bigger cells. Patch size significantly affects performance. A smaller patch size will fail to segment big cells, in contrast a bigger patch size diminishes the vq-vae and segmentation accuracy. In the case of the neuroblastoma dataset there are a few cells four or five times larger than typical ones. The current implementation does not handle these large cells since increasing the patch size so much will diminish the precision for the average cell. We plan to address this issue in future work.

We approach this article as a proof of concept, we are not trying to achieve sota performance. We did not implement any approach to improve handling of different patch sizes.

1.3 The only data preprocessing performed was to normalize the images. No data augmentation was applied. We have updated section 2.6 with this information.

1.4 The idea behind the 7x7 kernel is two fold: Avoid predicting isolated noisy pixels and avoid predicting exactly at the cell's boundary. The kernel parameter was selected by tests on the validation set of the U2OS dataset, but differences were minimal to slight changes in the parameter. The parameter is the same for both datasets.

The rationale for selecting ten points for U2OS and one for Neuroblastoma datasets is related to the convergence of the algorithm (see also 1.5 response), and memory-speed tradeoff. For the USO2 dataset keeping the parameter high helps the algorithm to not stop before predicting almost all cells in image and perform faster. However, increasing the parameter for the Neuroblastoma dataset

might cause memory problems since the patches are bigger and parallel processing increases memory consumption. Increasing the parameter in this case did not improve convergence, thus we decided to keep it at one .

1.5 The algorithm time is proportional to the number of cells in the image. For empty images is almost instantaneous since it will only convolve a kernel of ones along the image and try to do a limited number of predictions. Thus the algorithm is very fast for very big images with low density of cells.

In general, very high density of cells, noisy images with a lot of artifacts, and difficult to predict cells for the model will increase the computational time, but in these cases it seems reasonable to spend more computation.

See attached figure with the time Kaizen takes to process an image respect to the number of nuclei in the image for the U2OS dataset

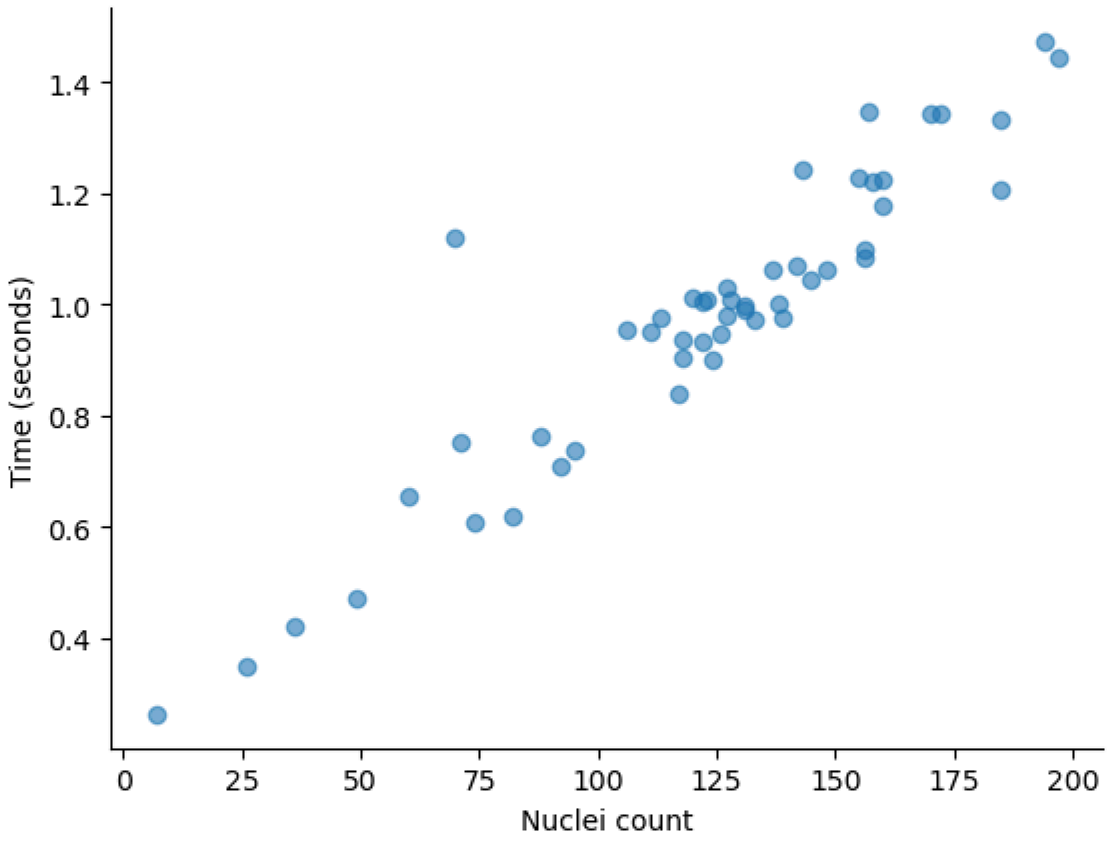

One advantage of the algorithm is a smaller memory requirement than other methods, since at any given time only part of the image is processed through the network.

We have considered adding a figure to the manuscript about this topic and discussing it. However, we thought that perhaps it is not a critical topic and we did not want to distract from the main message. Nevertheless, we are open to suggestions on tackling this topic in the manuscript.

2 We have added to Figures 3 and 4 outputs from other methods as suggested, for visual comparison.

3 The issue about reproducibility by other researchers was also raised by the other reviewer. We have decided to make the code available to facilitate reproducibility by other researchers. We are still working on refactoring the code since it was very messy. We will make everything available in github (https://github.com/Danielmaj/Kaizen) and in one of the repositories recommended by PLOS ONE.
